# Older Couples’ Marital Quality and Health Behaviors

**DOI:** 10.1093/geroni/igaa057.1118

**Published:** 2020-12-16

**Authors:** Angela Curl, Jennifer Bulanda, Amy Restorick Roberts

**Affiliations:** Miami University, Oxford, Ohio, United States

## Abstract

Health benefits of marriage may stem in part from spouses discouraging unhealthy behavior and encouraging healthy practices. Although studies show spousal effects on health behaviors, few have assessed whether spousal effects vary by the quality of the marital relationship. Spouses in low-quality marriages may be less likely to engage in joint activities that promote health (e.g., shared exercise), make fewer attempts at monitoring their spouse’s health behaviors, and be less successful in their attempts to intervene. Those in unhappy relationships may also use unhealthy behaviors as maladaptive coping strategies to deal with marital stress. We use dyadic data from couples over age 50 in the 2006 and 2008 waves of the Health and Retirement Study (HRS) to examine how both spouses’ ratings of positive and negative dimensions of marital quality are associated with their own and their spouses’ exercise and smoking (n=3,498 couples). Using HLM software, we estimated actor-partner interdependence models (APIM). Results indicate that both own and husbands’ ratings of positive marital quality are significantly associated with wives’ odds of smoking. Own perceptions of negative marital quality and wives’ perceptions of both positive and negative marital quality are associated with husbands’ odds of smoking. For wives, neither own nor spousal marital quality is significantly related to exercise. For husbands, however, wives’ higher positive marital quality and lower negative marital quality are associated with increased exercise. Strategies to improve marital quality may promote healthy behaviors among older adults, particularly for husbands.

